# Study protocol for the multicentre cohorts of Zika virus infection in pregnant women, infants, and acute clinical cases in Latin America and the Caribbean: the ZIKAlliance consortium

**DOI:** 10.1186/s12879-019-4685-9

**Published:** 2019-12-26

**Authors:** Vivian I. Avelino-Silva, Philippe Mayaud, Adriana Tami, Maria C. Miranda, Kerstin D. Rosenberger, Neal Alexander, Luis Nacul, Aluisio Segurado, Moritz Pohl, Sarah Bethencourt, Luis A. Villar, Isabelle F. T. Viana, Renata Rabello, Carmen Soria, Silvia P. Salgado, Eduardo Gotuzzo, María G. Guzmán, Pedro A. Martínez, Hugo López-Gatell, Jennifer Hegewisch-Taylor, Victor H. Borja-Aburto, Cesar Gonzalez, Eduardo M. Netto, Paola M. Saba Villarroel, Bruno Hoen, Patrícia Brasil, Ernesto T. A. Marques, Barry Rockx, Marion Koopmans, Xavier de Lamballerie, Thomas Jaenisch, the ZIKAlliance Clinical Study Group, Thomas Jaenisch, Kerstin Daniela Rosenberger, Ivonne Morales, Frank Tobian, Lorenz Uhlmann, Moritz Pohl, Julius Schretzmann, Annika Leege, Ernesto T. A. Marques, Isabelle F. T. Viana, Roberto D. Lins, Patrícia Brasil, Ana Maria Bispo de Filippis, Ana Claudia Machado Duarte, Otavio de Melo Espíndola, Myrna Bonaldo, Renata Rabello, Luana Damasceno, Vivian Avelino-Silva, Aluisio Segurado, Ester Sabino, Maria Cassia Mendes-Correa, Luis Nacul, Neal Alexander, Eduardo Martins Netto, Adriana Tami, Sarah Bethencourt, Cristel Falcon, Egri Rodríguez, Victmar Matos, Maria José Tinedo, Yenifer La Rosa, Marianela Murillo, Luis Angel Villar, Maria Consuelo Miranda, Anyela Lozano, Victor Mauricio Herrera, Adriana Gomez, Rosa Margarita Gelvez, Ricardo Ortiz, Carmen Soria, Lady Dimitrakis, Silvia Paola Salgado, Mary Regato Arrata, Eduardo Gotuzzo, Humberto Guerra Allison, Michael Talledo, Paola Mariela Saba Villarroel, Eric Martínez Torres, María G. Guzmán, Pedro A. Martínez Rodríguez, Mayling Alvarez Vera, Belkis Galindo Santana, Alicia Reyes, Silvia Serrano Álvarez, Diana Ferriol Dorticós, Jennifer Hegewisch-Taylor, Celia Alpuche-Aranda, Hugo López-Gatell, Esteban González-Diaz, Norma Pavía-Ruz, Victor Hugo Borja-Aburto, Cesar Gonzalez, Concepción Grajales, Teresita Rojas, Lumumba Arriaga, Alfonso Vallejos, Bruno Hoen, Dominique Tynevez, Xavier de Lamballerie, Laurence Thirion, Jan Felix Drexler, Barry Rockx, Eric van Gorp, Marion Koopmans

**Affiliations:** 1 Department of Infectious and Parasitic Diseases and Instituto de Medicina Tropical, Faculdade de Medicina da Universidade de Sao Paulo, Sao Paulo, Brazil; 2 Hospital Sirio-Libanes, Sao Paulo, Brazil; 3 London School of Hygiene and Tropical Medicine, London, UK; 4 Department of Medical Microbiology, University of Groningen, University Medical Center Groningen, Groningen, The Netherlands; 5 Facultad de Ciencias de la Salud, Universidad de Carabobo, Valencia, Venezuela; 6 Universidad Industrial de Santander, Bucaramanga, Colombia; 7 Department of Infectious Diseases, Section Clinical Tropical Medicine, Heidelberg University Hospital, INF 324, 69120 Heidelberg, Germany; 8 German Centre for Infection Research (DZIF), Heidelberg Site, Cologne, Germany; 9 Institute of Medical Biometry and Informatic, Heidelberg University Hospital, Heidelberg, Germany; 10 Aggeu Magalhães Institute, Oswaldo Cruz Foundation (FIOCRUZ), Recife, Brazil; 11 Evandro Chagas National Institute of Infectious Diseases, Oswaldo Cruz Foundation (FIOCRUZ), Rio de Janeiro, Brazil; 12 Universidad Católica Santiago de Guayaquil, Guayaquil, Ecuador; 13 SOSECALI C., Ltda, Guayaquil, Ecuador; 14 Instituto Nacional de Investigación en Salud Pública “Dr. Leopoldo Izquieta Pérez”, (INSPI), Guayaquil, Ecuador; 15 Instituto de Medicina Tropical Alexander von Humboldt, Universidad Peruana Cayetano Heredia, Lima, Peru; 16 Pedro Kouri Tropical Medicine Institute (IPK), Havana, Cuba; 17 Centro de Investigación sobre Enfermedades Infecciosas, Instituto Nacional de Salud Pública, Cuernavaca, Mexico; 18 Mexican Institute of Social Security, Mexico City, Mexico; 19 Federal University of Bahia, Salvador, Brazil; 20 Centro Nacional de Enfermedades Tropicales (CENETROP), Santa Cruz, de la Sierra, Bolivia; 21 INSERM Centre d’Investigation Clinique 1424, Centre Hospitalier Universitaire de Pointe-à-Pitre, Pointe-à-Pitre, France; 22 Service de Maladies Infectieuses et Tropicales, Dermatologie, Médecine Interne, Centre Hospitalier Universitaire de Pointe-à-Pitre, Pointe-à-Pitre, France; 23 Université des Antilles et de la Guyane, Faculté de Médecine Hyacinthe Bastaraud, 4537 Pointe-à-Pitre, EA France; 24 Department of Viroscience, WHO CC Arbovirus and hemorrhagic fever viruses reference and research, Erasmus MC, Rotterdam, The Netherlands; 25 Aix-Marseille University, Marseille, France; 26 University of Pittsburgh, Center for Vaccine Research, Pittsburgh, PA USA

**Keywords:** Zika, Pregnant women, Children, Cohort, Latin America, Caribbean, Risk, Congenital abnormalities, Natural history, Arboviruses, Mosquito-borne viruses, Vector-borne viruses

## Abstract

**Background:**

The European Commission (EC) Horizon 2020 (H2020)-funded ZIKAlliance Consortium designed a multicentre study including pregnant women (PW), children (CH) and natural history (NH) cohorts. Clinical sites were selected over a wide geographic range within Latin America and the Caribbean, taking into account the dynamic course of the ZIKV epidemic.

**Methods:**

Recruitment to the PW cohort will take place in antenatal care clinics. PW will be enrolled regardless of symptoms and followed over the course of pregnancy, approximately every 4 weeks. PW will be revisited at delivery (or after miscarriage/abortion) to assess birth outcomes, including microcephaly and other congenital abnormalities according to the evolving definition of congenital Zika syndrome (CZS).

After birth, children will be followed for 2 years in the CH cohort. Follow-up visits are scheduled at ages 1–3, 4–6, 12, and 24 months to assess neurocognitive and developmental milestones. In addition, a NH cohort for the characterization of symptomatic rash/fever illness was designed, including follow-up to capture persisting health problems.

Blood, urine, and other biological materials will be collected, and tested for ZIKV and other relevant arboviral diseases (dengue, chikungunya, yellow fever) using RT-PCR or serological methods. A virtual, decentralized biobank will be created. Reciprocal clinical monitoring has been established between partner sites.

Substudies of ZIKV seroprevalence, transmission clustering, disabilities and health economics, viral kinetics, the potential role of antibody enhancement, and co-infections will be linked to the cohort studies.

**Discussion:**

Results of these large cohort studies will provide better risk estimates for birth defects and other developmental abnormalities associated with ZIKV infection including possible co-factors for the variability of risk estimates between other countries and regions. Additional outcomes include incidence and transmission estimates of ZIKV during and after pregnancy, characterization of short and long-term clinical course following infection and viral kinetics of ZIKV.

**Study registrations:**

clinicaltrials.gov NCT03188731 (PW cohort), June 15, 2017; clinicaltrials.gov NCT03393286 (CH cohort), January 8, 2018; clinicaltrials.gov NCT03204409 (NH cohort), July 2, 2017.

## Background

Identified in the 1950’s [1–3], Zika virus (ZIKV) emerged as a critical public health issue only in 2015, when it caused a large epidemic in Latin America and the Caribbean that brought previously overlooked complications to light [4–8]. Clusters of microcephaly and neurological manifestations observed in late 2015, together with recognized widespread ZIKV activity in Brazil [9–12], prompted the World Health Organization (WHO) to declare a Public Health Emergency of International Concern from the 1st February until the 18th November 2016 [13]. Despite the numerous scientific publications on ZIKV infection produced in the past years, crucial information on the natural history as well as on the definition and precise risk quantification of congenital Zika syndrome (CZS) and other developmental abnormalities are still lacking, and well characterized prospective cohort studies are needed to clarify these.

**Fig. 1:**
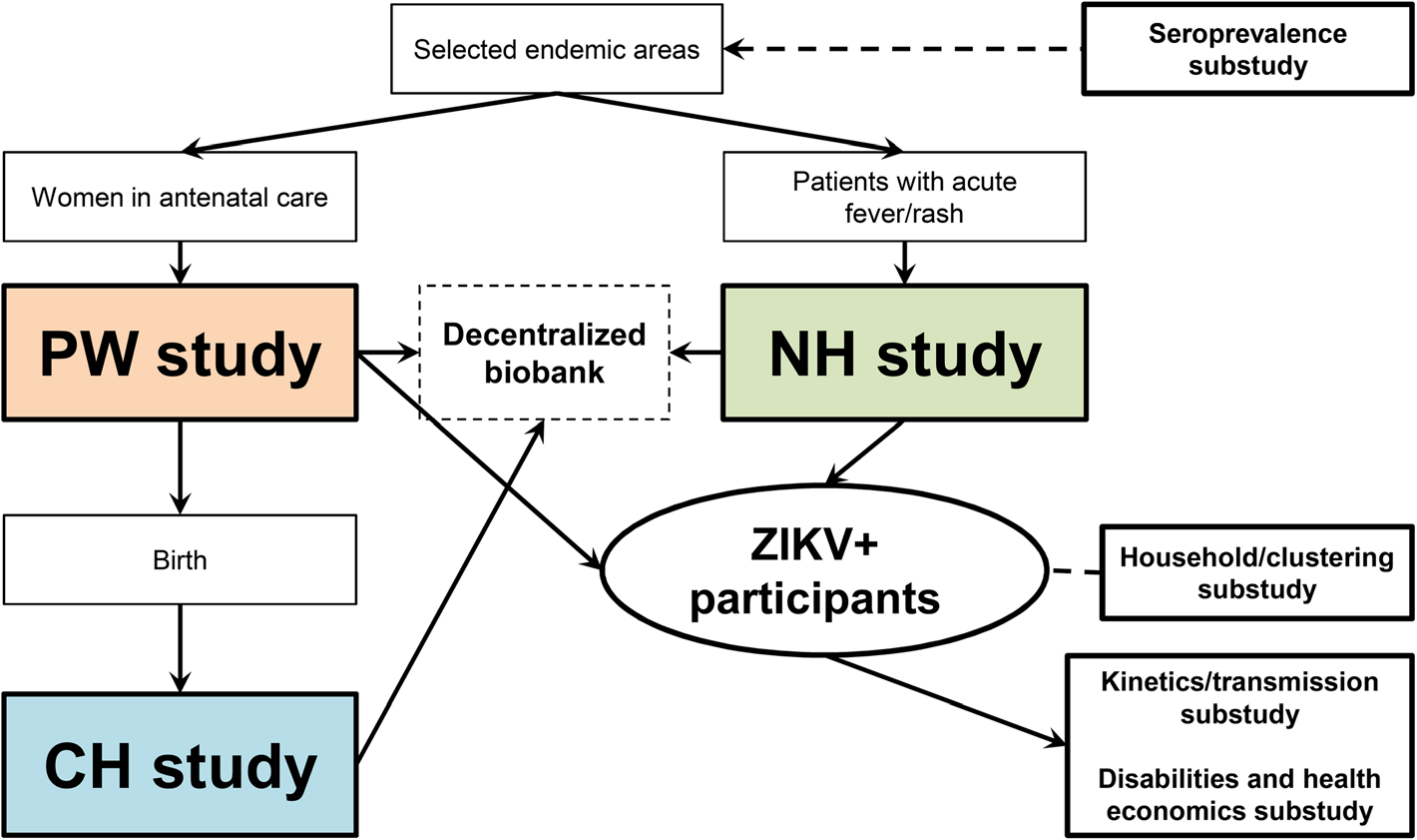
ZIKAlliance clinical cohorts: Pregnant Women (PW) cohort, Children (CH) cohort, and Natural History (NH) cohorts, including connected substudies and decentralized biobank

Here we report the design of the prospective multicentre observational cohort studies conducted within the European Commission (EC) Horizon 2020 (H2020)-funded ZIKAlliance Consortium (https://zikalliance.tghn.org/). The Consortium as a whole includes 53 partners and is organized in eight scientific and one management work packages (WPs), as well as three cross-cutting WPs, which have been added to organize collaboration with the other EC H2020-funded consortia: ZikaPlan (https://zikaplan.tghn.org) and ZikAction (http://zikaction.org/). The cross-cutting WPs relate to the harmonization of protocols and data sharing, to common management and communication strategy, and to the organization of a joint preparedness network in Latin America and the Caribbean. In this manuscript, we focus on the design of the clinical observational studies comprising the “Clinical Sciences” (WP1) and “Clinical Biology and Immunology” (WP2) WPs. These include a Pregnant Women (PW) cohort, a Children (CH) cohort, a Natural History (NH) cohort, and a number of connected substudies (Fig. 1).

With this integrated prospective research approach, we aim to i) quantify the risk of complications in CH born to mothers infected during pregnancy, ii) identify risk factors for these complications, and iii) describe the NH of infection, disease and development in these CH as well as iv) in adults. Clinical sites have been selected over a wide geographic region bearing in mind the evolving course of the ZIKV epidemic, allowing flexibility in enrolment by shifting towards areas with new or ongoing ZIKV transmission. The network of clinical sites builds on pre-existing networks for dengue virus (DENV) studies [14], with the goal to expand to a broad platform of collaborations and institutional partnerships between Europe, Latin America and the Caribbean dedicated to emerging diseases preparedness research. Here, we describe the main study designs and the common diagnostic and biobanking approach.

## Methods

### Study sites

**Fig. 2:**
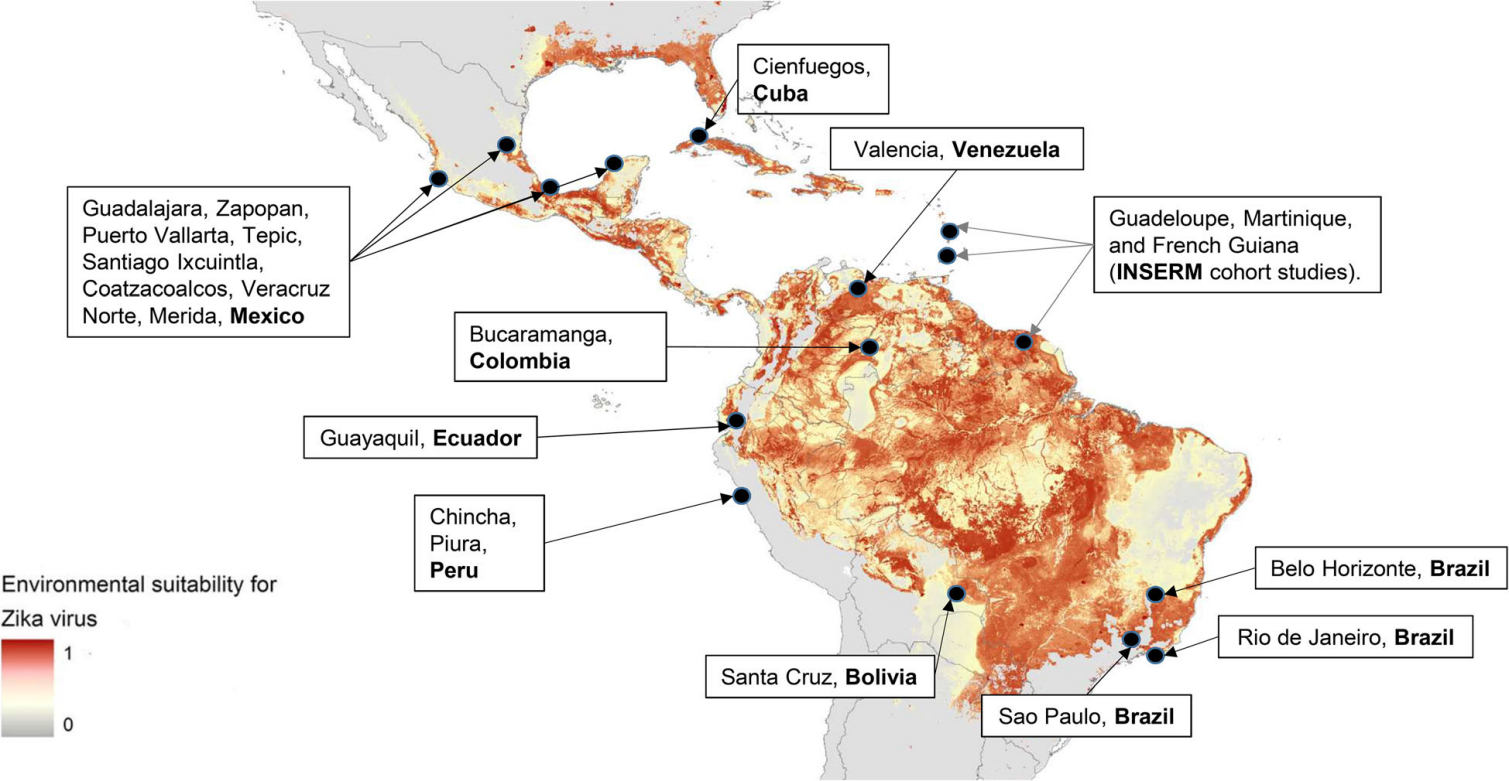
ZIKAlliance study sites for the pregnant women (PW), and children (CH) cohorts (map showing environmental suitability of Zika virus transmission according to Messina et al., 2016 [15], modified with inclusion of ZIKAlliance study sites)

The PW and CH cohort studies are carried out in selected study sites across Latin America and the Caribbean (Fig. 2). Study sites are selected based on a) existing or recent ZIKV transmission, combined with ongoing collaborative research (for example the EC-funded IDAMS-Dengue study [14]); and b) on the projection of the fringes of the mosquito vectors distribution [15]. The latter is aimed at including locations still vulnerable for future outbreaks of ZIKV in Latin America at the time of the start of the study in 2016/17. Wherever available, community seroprevalence surveys are used to target regions where ongoing transmission is likely [16–18].

**Fig. 3:**
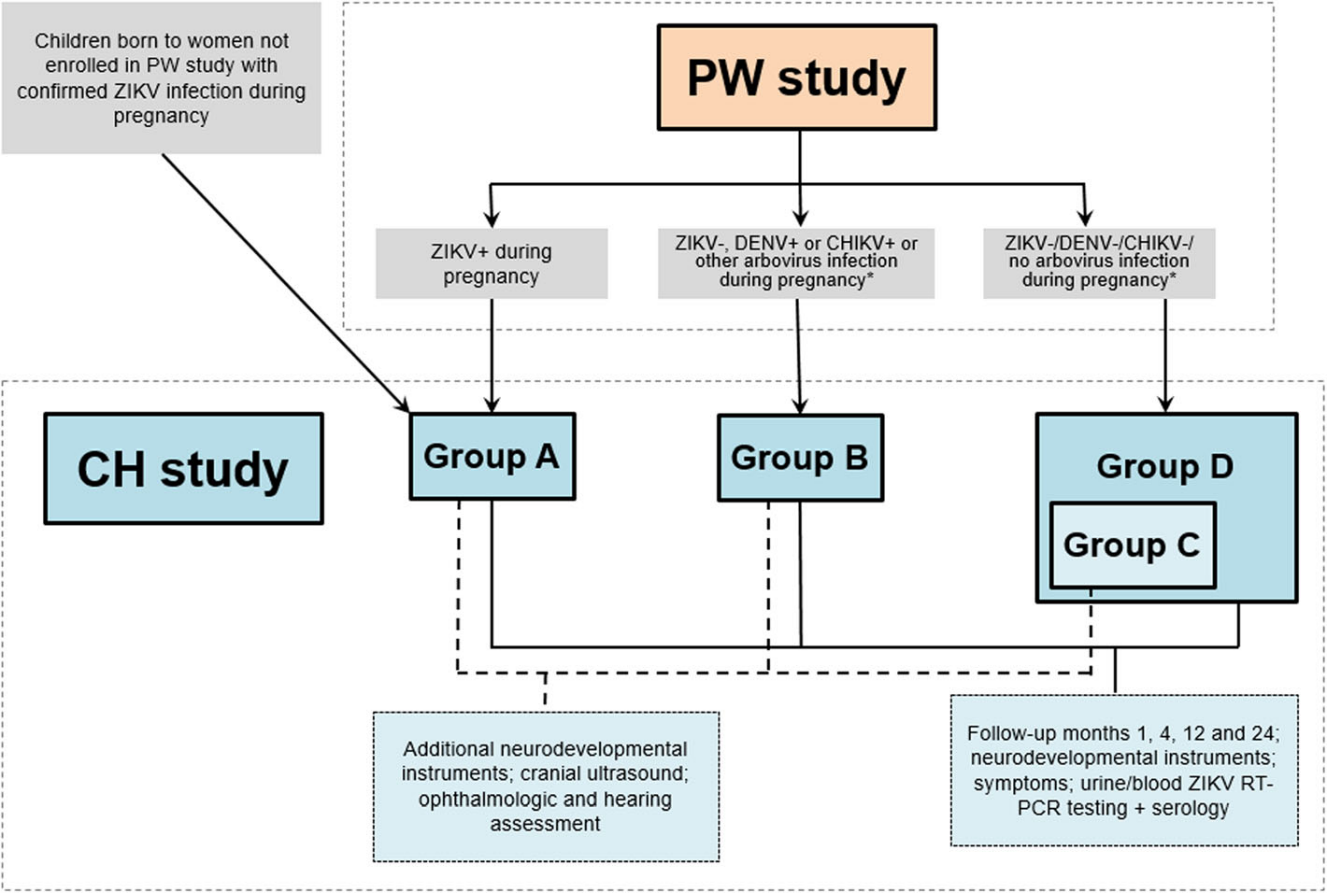
ZIKV birth cohort comprising pregnant women (PW) and children (CH) cohort studies

### Pregnant women (PW) cohort study

Strong evidence has been produced for a causal relationship between ZIKV infection during pregnancy and microcephaly of the baby (as well as a range of other congenital anomalies) through clinical-epidemiologic studies [8, 19–21], histopathological and virologic demonstration of ZIKV in affected tissues [22–24], and supportive evidence from in vitro studies and animal infection models [25–28]. However, the quantification of this relationship has proven difficult to measure. Co-factors or effect modifiers have been suggested to be of importance [29–31], but their exact role has not been adequately measured.

We aim to estimate the absolute and relative risk of ZIKV-associated congenital abnormalities and evaluate the role of co-factors or effect modifiers responsible for the variability of the risk currently observed across Latin America and the Caribbean, including previous exposure to other arboviruses. This will require strong support from the laboratories linked to the enrolling sites, in order to develop the expertise and tools needed to diagnose and monitor maternal, foetal and neonatal infections with high sensitivity and specificity.

**Box 1** Key Information of the ZIKAlliance Pregnant Women (PW) cohort study

**Table:** 

**Pregnant Women (PW) cohort (****clinicaltrials.gov** **identifier: NCT03188731)** **Primary Objectives** 1. To estimate the absolute and relative risks of congenital abnormalities and adverse pregnancy outcomes associated with Zika virus (ZIKV) infection during pregnancy 2. To describe the spectrum of abnormalities and adverse pregnancy outcomes associated with ZIKV infection during pregnancy, further characterizing the congenital Zika syndrome (CZS) **Secondary Objectives** 1. To improve the diagnosis of ZIKV infection in PW and neonates 2. To measure the incidence and risk factors of ZIKV infection in PW across a number of sites in Latin America and the Caribbean 3. To describe the clinical spectrum of ZIKV infection during pregnancy, and determine the proportion of symptomatic and asymptomatic infections 4. To determine the rate and risk factors of mother-to-child transmission of ZIKV (cross-cutting objective with the children [CH] cohort [see below]) 5. To evaluate the role of co-factors or effect modifiers affecting the variability of current risk estimates for congenital abnormalities 6. To compare the risk of infant abnormalities and other adverse outcomes between symptomatic and asymptomatic PW 7. To measure the association between time of ZIKV infection during pregnancy and resulting abnormalities in the foetus or other pregnancy outcomes **Inclusion/exclusion** Inclusion criteria • Age ≥ 16 years old • Confirmed pregnancy • Gestational age ≤ 27 weeks (extended to ≤32 weeks for inclusion in the first month of transmission at a location with marked transmission) • Available and willing to undergo study visits and procedures • Written informed consent (assent form and consent by a legal guardian if applicable) Exclusion criteria • Participation in another PW cohort study concerning ZIKV **Endpoints** • Congenital abnormality or adverse pregnancy outcome (according to the evolving definition of the CZS)	

#### Study design

Screening of PW will take place in routine antenatal care clinics in selected sites in Latin America and the Caribbean. PW will be enrolled regardless of ZIKV symptoms at baseline.

PW will be followed over the course of the pregnancy approximately every 4 weeks making use of routine antenatal care visits and revisited at delivery (or after miscarriage/abortion) with assessment of rash/fever illness episodes and collection of serial blood/urine samples. Investigations will also include a comprehensive assessment of potential co-factors or effect modifiers for the risk of congenital malformations in ZIKV-infected PW, such as demographics, socio-economic status, living conditions, TORCHS infections (Toxoplasmosis, Rubella, Cytomegalovirus, Herpes and Syphilis; to be tested in a subset and according to national guidelines), HIV serostatus, previous flavivirus infections or vaccines (e.g. DENV or yellow fever [YF]), environmental toxins, and chromosomal abnormalities. Symptomatic infections with other arboviruses (including, but not limited to DENV, Chikungunya [CHIKV], YF, Mayaro, Oropouche) during pregnancy will also be evaluated as possible co-factors. PW with suspected or confirmed ZIKV infection will be managed according to national protocols.

The visit at birth will be considered the last visit in the PW cohort and the starting point for the CH cohort. Samples from placenta, amniotic fluid, cord blood, maternal blood and urine and blood from the infant will be collected at the birth visit. All newborns will receive a detailed examination. Any abnormalities will be recorded and evaluated according to the evolving definition of the CZS [32]. Laboratory-confirmed ZIKV infection will be defined based on a positive ZIKV quantitative reverse transcriptase (RT)-PCR in either body fluid or tissue biopsy (e.G. *placenta*). Seroconversion between paired samples collected at different points of time may be considered as a possible recent ZIKV infection. In view of the conflicting evidence on the specificity of currently available serological assays, the final stratification will be based on an exhaustive evaluation of a subset of PW, which will include the use of (combinations of) assays developed to increase discriminatory power for flavivirus antibody testing [33, 34]. Over the course of this study, both laboratory and clinical diagnostic criteria may undergo modifications based on results from this study and others.

### Children (CH) cohort study

Infants with in utero ZIKV exposure may present with variable degrees of abnormalities, ranging from unapparent infection to a recognizable pattern of structural anomalies and functional disabilities, most of them due to nervous system damage [19, 35–37]. However, a normal examination at birth does not guarantee normal development. Post-natal ocular abnormalities have been reported in infants born with and without microcephaly [38, 39]. Late-term neurologic and cognitive abnormalities for these infants are considered to be likely, but are largely unknown. Moreover, it is unclear whether perinatal and early post-natal infections could also lead to complications. This is suggested by the description of immune-mediated neurological complications among infants infected postnatally and abnormalities among infants whose mothers were exposed to ZIKV in the third trimester of pregnancy [40, 41].

We aim to determine the absolute and relative risks of developmental and neurological abnormalities in the first 2 years of life in CH born to women infected with ZIKV during pregnancy.

**Box 2** Key information of the ZIKAlliance Children (CH) cohort study

**Table:** 

**Children cohort (CH;** **clinicaltrials.gov** **identifier: NCT03393286)** **Primary Objectives** 1. To estimate the absolute and relative risks of developmental, neurological, ophthalmic, and auditory abnormalities during the first 2 years of life in children born to women infected with Zika virus (ZIKV) during pregnancy compared to women not infected during pregnancy 2. To describe the spectrum of abnormalities and clinical course during the first 2 years of life in children born to women with ZIKV, dengue virus (DENV) Chikungunya (CHIKV) or possibly other arbovirus infections during pregnancy **Secondary Objectives** 1. To evaluate risk factors for developmental abnormalities in children exposed to ZIKV during pregnancy 2. To describe the incidence of ZIKV infections after birth in early childhood and evaluate the associated risks for abnormal child development; 3. To determine the incidence of ZIKV infection in the mother after birth and evaluate its clinical features as well as its kinetics in different body fluids 4. To determine the rate and risk factors and effect modifiers for congenital, perinatal and postnatal mother-to-child transmission of ZIKV 5. To describe the kinetics and evolution of infection and immune responses to ZIKV infection in infants and children, stratified by transmission pattern (for example in utero vs. post-natal) **Inclusion/exclusion** (Fig. 3) Inclusion criteria • Group A: Infants born to mothers with confirmed ZIKV infection during pregnancy • Group B: Infants born to mothers with confirmed DENV, CHIKV or other arboviral infection during pregnancy (detected in incident fever/rash episodes) • Group C: Random sample of infants born to mothers without ZIKV/DENV/CHIKV or another arboviral infection during pregnancy (from ZIKAlliance PW cohort), 1:1 proportion compared to groups A and B together. The remaining children from mothers who were not infected with any of the three arboviruses are followed in Group D (standard visitation schedule) • Mother-infant pairs available to undergo study visits and procedures • Written informed consent (assent form and consent by a legal guardian if applicable) Exclusion criteria • Mother aged below 16 years old **Endpoints** • Infant developmental abnormalities as assessed by neurological examination, Ages & Stages Questionnaire® and Bayley Scales of Infant Development	

#### Study design

CH of women exposed to ZIKV during pregnancy, and a subset of those not exposed, will be followed after birth for developmental milestones (neuro-cognitive, motor, ophthalmic, and auditory defects) and also to assess the risk of post-natal ZIKV infection among susceptible infants. We will also follow CH of women with incident arboviral infection (including DENV, CHIKV or other such as YF) detected in symptomatic fever/rash episodes over the course of the pregnancy.

At delivery (last visit of PW study), investigations of the mother will include a full blood count, serological tests for ZIKV as well as RT-PCR-testing in blood and urine for ZIKV. The presence of potential environmental pollutants will be tested in a subsample of PW from appropriate biological materials. In addition, placenta and umbilical cord tissue, cord blood, and amniotic fluid will be collected. All biological material will be collected and stored according to detailed SOP. TORCHS and HIV testing will be carried out according to national guidelines and, if not available, may be performed in stored samples for the purposes of the study.

The investigations performed on the infant at birth will include a standard neonatal examination comprised of Apgar scores, anthropometry, reflexes and muscle tone, and collection of blood via heel prick or venepuncture if necessary. Screening tests will be carried out at birth according to national guidelines. Additional investigations, such as cerebral imaging or collection of cerebrospinal fluid, will be carried out only if medically indicated.

Follow-up visits will be scheduled at ages 1–3, 4–6, 12, and 24 months to assess the infant’s development using comprehensive neuro-developmental evaluation instruments (Ages & Stages Questionnaire [ASQ-3] and Bayley Scales of Infant Development) [42, 43]. Blood and urine samples from the infant will be collected to test for ZIKV infection (using RT-PCR) or seroconversion. In the mother, we will also test blood and urine samples, with added testing of saliva and breast milk in breastfeeding women who test RT-PCR-positive in urine or blood. CH with any abnormalities detected at birth or during follow-up will be managed according to national protocols.

### Natural history (NH) cohort study

The ZIKV epidemic in Latin America and the Caribbean occurred in parallel or following a wave of CHIKV and in the context of a high DENV incidence. Given the high frequency of co-circulating arboviruses and their overlapping clinical features, the correct diagnosis of ZIKV infection based on clinical features and antibody testing is challenging, leading to inaccuracies in notifications [44], and potentially impairing the implementation of effective public health interventions. The current case definition for symptomatic ZIKV infections is broad [45] and its performance has not been empirically validated. ZIKV infection may frequently be asymptomatic or associated with mild symptoms including low-grade fever, itchy skin rash, conjunctivitis, arthralgia and myalgia starting 3 to 12 days after infection; asymptomatic infection can occur in up to 80% of individuals [8, 46–49]. Neurologic complications including Guillain-Barré syndrome, myelitis and encephalitis [6, 11, 12, 50–53] have been reported following ZIKV infection [30, 31] in a frequency of around 1 to 5 per 10,000 adults [12].

Therefore, in the NH study, patients aged 5 years old and older in ZIKV-endemic areas presenting to healthcare units with acute fever/rash illness or any other biological evidence for ongoing ZIKV infection will be enrolled with baseline PCR testing for ZIKV and other co-circulating arboviruses depending on the local epidemiology (including, but not limited to, DENV and CHIKV). They will be followed daily over the acute illness period in order to obtain a detailed description of the clinical features, leading to validated case definitions. In addition, we will include phone interviews over the first year following acute infection, in order to evaluate the incidence of complications.

Nested into the NH study will be substudies focusing on the transmission, viral kinetics and shedding, antibody responses, and long-term disease burden of ZIKV infection. Symptomatic PW from the PW cohort will also contribute to the knowledge base of the NH study.

The aim of the NH cohort study is to generate evidence for a more precise clinical case definition for ZIKV infections in the context of co-circulating arboviral infections like DENV, CHIKV or YF (vaccination) and to better evaluate the full clinical spectrum of ZIKV infections. The enhanced clinical case definition will be evaluated against the current candidate WHO case definition for ZIKV infection [54].

**Box 3** Key information of the ZIKAllliance Natural History (NH) cohort study

**Table:** 

**Natural History (NH) cohort (****clinicaltrials.gov** **identifier: NCT03204409)** **Primary Objectives** 1. To describe the clinical spectrum of disease manifestations among individuals with febrile illness or rash (including medium-term follow-up) and produce a validated case definition of Zika virus (ZIKV) disease **Secondary Objectives (including substudies)** 1. To improve the case definition of ZIKV disease 2. To establish a precise biological description of the natural course of the infection, with special emphasis on viral kinetics in body fluids (Household and Viral Kinetics substudies) 3. To determine the incidence of complications and long-term sequelae, including severe disease presentations, chronic fatigue, ophthalmologic manifestations, depression and other disabilities (Disability Substudy); 4. To investigate the socio-economic impact associated with ZIKV infection (Health Economics Substudy) 5. To describe the levels of ZIKV exposure in different epidemiological contexts **Inclusion / exclusion** Inclusion criteria • Age ≥ 5 years • Fever (≥37.5 °C) and/or rash for ≤5 days • Available to undergo study visits and procedures • Written informed consent (assent form and consent by a legal guardian if applicable) Exclusion criteria • Localizing features suggesting an alternative diagnosis, e.g. pneumonia, otitis media **Endpoints** • Clinical characterisation (signs/symptoms) and consequences of ZIKV, dengue virus (DENV), Chikungunya (CHIKV) and possibly other arboviral diseases • Occurrence and duration of viral ZIKV shedding in different biologic fluids • Incidence of ZIKV-associated complications and disability • Prevalence, incidence and risk factors of ZIKV among individuals with high or low risk of potential sexual transmission, as well as among household contacts of index patients.	

#### Study design

Participant enrolment for the NH study will take place in primary care and emergency units in selected sites. Patients aged ≥5 years old presenting with acute febrile/rash illness in the last 5 days, and without overt signs/symptoms suggesting alternative diagnosis will be invited to participate.

At enrolment, demographic and clinical information will be collected, with detailed assessment of signs/symptoms currently included in the WHO case definition of Zika virus disease [54], as well as symptoms of infection with other co-circulating arboviruses such as (but not limited to) DENV or CHIKV, and basic haematology and biochemistry assessments. We will also collect early blood samples during the acute phase of illness for PCR testing of the relevant etiological agent (concentrating on the above-mentioned arboviruses). We will strive to detect additional infectious agents and hence improve the characterization of the clinical phenotype of ZIKV infection compared to other pathogens. Participants will be followed daily (including laboratory investigations) for 2–4 days, with an additional visit at 14 days after enrolment to evaluate the clinical phenotype of the acute illness period and obtain a convalescent blood sample for paired testing. This will also allow the documentation of atypical clinical phenotypes or complicated clinical presentations.

Patients will be contacted by phone over the following year to capture persisting complaints or late complications. Medical management will be carried out according to national guidelines. We will analyse the spectrum of clinical manifestations stratified by age, prior flavivirus immune status, and presence of comorbidities.

### Substudies

Substudies of ZIKV viral kinetics, clustering of ZIKV transmission, impact on disability and health economics, the potential role of antibody enhancement, and co-infections will be connected to the NH and PW cohort studies (Fig. 1). ZIKV transmission occurs mainly through the bite of an infected female *Aedes* mosquito [47], but transmission through blood transfusion [55–57], sexual intercourse [58–65] and direct contact through intact skin [66] have also been reported. ZIKV shedding seems to occur in a range of body fluids, most notably in male genital fluids where shedding can be protracted for months [64, 67]. Some reports also suggest shedding can also be prolonged in PW [27, 68, 69]. The frequency and duration of ZIKV shedding in different body fluids is unknown, as is the significance of viral shedding for the risk of person-to-person transmission.

#### Seroprevalence studies in communities

Estimates of ZIKV incidence in affected areas are often based on notifications with insufficient laboratory confirmation resulting in misclassifications due to other circulating arboviruses [44]. Seroprevalence studies in communities representing the epidemiological context of potential participants will be underway to guide the selection and potential relocation of sites for ZIKAlliance cohorts over the course of the study. Populations under investigation include groups representing a range of socioeconomic status, environmental exposure and susceptibility to viral sexual transmission.

#### Viral kinetics substudy

This substudy will enrol adults with PCR-confirmed ZIKV identified in the cohorts in selected sites who consent with a more intense follow-up including genital samples if possible. Scheduled visits occur at days 7, 14, 21 and 28 after inclusion, then months 3, 6 and 12, with additional collection of samples until no ZIKV is detected in any sample. The primary outcome will be the frequency and duration of ZIKV detection in blood and other body fluids (saliva, urine, genital secretions, breast milk) for 12 months following acute ZIKV infection, and secondary outcomes will include potential associations with clinical manifestations and long-term complications or sequelae.

#### Household substudy

Household (HH) contacts of PW or NH participants with confirmed ZIKV infection will be invited to participate in in selected study sites. The main objective is to compare the prevalence of ZIKV infection in well-defined groups, using RT-PCR in blood and urine samples, as well as serological methods. Of special interest is the ratio of the markers of ZIKV infection between sexual partners compared to other HH contacts. In virologically-confirmed patients, we will conduct phylogenetic analysis to establish potential concordance of ZIKV strains within and between HH. HH contacts will be defined as any person aged 5 years and older spending > 1 week in the same HH within 1 month of the index patient being identified.

#### Disability and health economics substudies

We will describe medium-term complications and disabilities among ZIKV patients following acute infection, and the impact of ZIKV infection on health, quality of life and disability. We will estimate the socio-economic burden of the disease in terms of use of family resources, loss of income and leisure time.

### Laboratory support to studies

In order to ensure comparability of inclusions across the sites, protocols, facilities, and capacity for virologic and serological testing will be assessed for the laboratories linked to the clinical centres. Initial molecular testing as well as IgM/G serology will be done locally, with proficiency testing provided through the project. For molecular testing, locally available protocols will be used. Laboratories with insufficient performance in the External Quality Assurance (EQA) testing are supported by laboratory partners in the project for troubleshooting and confirmatory testing.

Molecular testing is the gold standard for ZIKV diagnosis and is definitive evidence for infection. Laboratory testing will be carried out using RT-PCR (in plasma/serum, urine, and other body fluids for ZIKV) and serology (serum/plasma). An initial inventory of essential diagnostic capacity of the laboratories associated with clinical sites was performed, based on a previously developed laboratory capacity and capability survey for ZIKV diagnostics [70, 71]. Standards have been prepared for molecular diagnostic testing [72, 73]. EQA for molecular detection of ZIKV was carried out at the start of the study, and will be repeated at regular intervals [74]. Should lack of sensitivity be noted, the site will be contacted, and test algorithms revisited, including changes in assays or protocols. In order to harmonize laboratory procedures, detailed standardized laboratory protocols were developed describing the sampling frame, sample volumes, shipping and storage conditions.

Several serological assays measuring anti-Zika virus IgM and IgG are commercially available. From the beginning of this study, all sites have been using such assays, for which sensitivity and specificity estimates have ranged from 88 to 95% and 30–98%, respectively [75]. The highest estimates were generated in targeted evaluation studies during the peak outbreak phase, and in regions with a lower background frequency of prior flavivirus exposure. The choice of the assays will be re-evaluated on a continuous basis and may be adapted according to new developments.

At enrolment, all participants of the PW cohort will be tested for IgG (ZIKV, DENV) and IgM (ZIKV) antibodies as well as via RT-PCR (as mentioned). Previous flavivirus infection status as well as vaccinations, if applicable (i.e. against YF or DENV) will be assessed as potential risk factors. In the case that other (arbo) viruses emerge in a certain location, they will be included in the testing panels. Positive PCR results will be repeated for confirmation including RNA extraction, unless both urine and blood are tested positive initially. IgG and IgM will be repeated at birth. Additional tests (e.g. serology) will be carried out either batched or individually, also depending on the laboratory capacities at each location. In the event of confirmed seroconversion at birth, retrospective testing of samples will be carried out for identification of timing of infection during pregnancy. Serologic tests will be performed using the approved, commercial kits and in the designated laboratories in each country. ZIKV IgM will be tested once every trimester of pregnancy as these antibodies may be short-lived. The best frequency for ZIKV IgM testing will still be subject to validation. In a subset of PW residing in an area with documented ZIKV transmission in the previous season with detectable anti-ZIKV IgG at enrolment, we will evaluate the specificity of the IgG assay by confirmatory virus neutralisation (VNT) assays with ZIKV and DENV and other ZIKV specific serological assays, performed in a reference laboratory. Results of the VNT and other assays may trigger adaptations of the follow-up schedule (e.g. for women who are no longer under risk of ZIKV infection).

Routine laboratory facilities in each participating site will be engaged in performing laboratory tests such as full blood count or biochemistry. TORCHS and HIV screening are part of the routine health investigations of PW in many countries. Where this is not available, TORCHS and HIV screening will be performed in samples from all mothers who have experienced adverse pregnancy outcomes or where congenital abnormalities are documented. In addition, a laboratory capacity strengthening program will be done in liaison with other consortia, and with an European network of diagnostic virology laboratories [70, 71, 73].

**Box 4** Diagnostics used in the ZIKAlliance studies

**Table:** 

**Diagnostics** **Diagnostic approach** The diagnostic approach is subjected to validation over the course of the study. The diagnostic approach includes: - ZIKV RT-PCR in plasma and urine at every visit for PW, CH and NH protocols - ZIKV IgM/IgG at PW enrolment and at birth (and at least one IgM every trimester of pregnancy) - ZIKV IgM/IgG at every visit of the CH cohort (both mother and child) and in paired samples in participants of the NH study - Dengue virus (DENV) IgG at enrolment in a subset (possibly using new platforms and/or VNT for the assessment of past infection status) - RT-PCR for DENV and CHIKV in addition to ZIKV in symptomatic participants **Harmonisation of diagnostics** An EQA for molecular detection of ZIKV was performed at the start of the study and will be repeated at regular intervals to assess the quality of the currently used molecular test systems for ZIKV detection in the ZIKAlliance diagnostic laboratories. This includes: - An inventory of essential diagnostic capacity in the laboratories associated with clinical sites, based on previously developed capacity, and a capability survey for ZIKV diagnostics - Detailed standardized laboratory protocols describing the sampling frame, sample volumes, shipping and storage conditions - Standards have been prepared for molecular diagnostic testing in the laboratories associated with clinical sites in order to assess sensitivity/specificity issues If issues in the precision or accuracy of diagnostic tests are noted, the site is contacted for revision of test algorithms, including changes in assays or protocols.	

### Data management and biobanking

Participants will be assured that all information generated in this study will remain confidential. All data (including clinical, laboratory and genetic data) will be stored in password-protected databases. Personal identifier information will be linked to stored data or samples only by a protected Master List (pseudonymization). This list will be kept under lock locally and will not be shared outside the study staff at the local partner site. No identifying information will be transferred between sites or to the central database. A governance board including members from all partners has been defined to coordinate the use and potential sharing of data and samples.

Electronic web-based case report forms (CRFs) have been developed using REDCap [76]. An electronic data entry interface was set up with the option of online data entry using a tablet device. Patient records will be entered as pseudonymized records (by use of a study identification number), not allowing the personal identification of patients. Clinical data and locally performed laboratory results will be entered directly to the electronic database. If initial data collection is performed in paper CRFs, data entry will be performed in a REDCap electronic CRF that mirrors the layout of the paper form.

Privacy and data safety will be ensured by a secured backup server and restricted access, also accommodating different levels of data access for participant identifiable information. Upon entry, data will automatically be validated across the entire patient record to fit the specific protocol requirements (plausibility and range checks). Data queries and source data verification requests will be generated and relayed back to each participating site for correction. A virtual, decentralized biobank will be created with an inventory of which samples will be available and where, how they will be stored, and what (raw) laboratory data will be associated. Samples will be stored locally, and a governing body created to ensure access and respond to requests for samples. The collection and storage of biological specimens will be regulated by SOP that are accessible to all partners.

### Data analysis

Because of the exploratory nature of the analysis, we did not conduct an exact sample size calculation. However, some power assessments were performed. For our purposes, we only consider microcephaly, because the variability with regard to the frequency of microcephaly is considerably smaller than that of other congenital abnormalities currently included in the CZS definition. Based on estimates of the Latin American Collaborative Study on Congenital Malformations (ECLAMC) [77], we assume a baseline risk of 0.025% for microcephaly in children born to ZIKV-uninfected mothers. For mothers ZIKV-infected during pregnancy, we assume a probability of microcephaly between 0.25 and 6% (relative risks between 10 and 240) [19, 20, 37, 78–81]. Furthermore, we assume that the probability of a ZIKV infection during pregnancy ranges between 5 and 30%. With a sample size between 5000 and 6000 we can detect, with 80% power and significance level α = 0.05, a relative risk of 20 when the probability of ZIKV infection is 10% or higher, and a relative risk of 30 when this probability is 5% or higher.

#### PW study

Data analysis will be carried out according to a pre-specified statistical analysis plan. The characteristics of the PW included will be assessed in a descriptive analysis, taking into account the grouping of the PW by ZIKV infection status. Possible differences between the infection groups will be detected by appropriate measures of location and corresponding tests (e.g. t-test, chi-square test, Wilcoxon rank-sum test). The primary endpoint will be any congenital abnormality or adverse pregnancy outcome, based on the emerging definition of the CZS [32]. Any congenital abnormality will be assessed at birth but may be updated if an abnormality only becomes apparent later in the neonatal period. In a first step, the primary endpoint will be analysed by a chi-square test. Next, a generalised mixed model will be fitted which contains predefined basic variables. The model will be investigated and modified by a stepwise back- and forward variable selection of the fixed effects. The region (represented by the local partner site or by a group of sites) will be included as a random effect in the model and will account for the heterogeneity between different regions. Sites may be aggregated based on geographical proximity in case of data sparseness. Interaction effects between two variables will only be included if there is a clinical rationale. We will present odds ratios as results of the generalised mixed model. Throughout the analyses, we will use α = 0.05 as significance level.

#### CH study

The primary endpoint in the CH study will be the detection of any developmental abnormality within 2 years of birth. The children in the study are born to women from the PW study or to mothers with proven ZIKV infection during pregnancy. The two-year follow-up brings up the practical restriction of drop-outs as right censoring. Consequently, the primary endpoint will be analysed with survival analysis techniques. Predefined basic variables on the outcome will be included in a Cox model with region as a random factor. Specific developmental abnormalities, which are only measured at one follow-up visit, will be evaluated by mixed logistic models, containing the respective variables, with region as a random effect as above.

#### NH study

The NH study will be carried out at a subset of the sites including participants who experience acute rash/fever illness, suggesting arboviral aetiology. The sample size will depend on the local epidemiology. The statistical analysis will be largely descriptive and informed by prior studies of arboviral rash and febrile illness [14, 82]. The prospective nature of the study will be used to describe the trajectory over time of signs and symptoms, and hence further define the case definitions of arbovirus infections, including ZIKV.

### Harmonisation, clinical monitoring, ethics, and dissemination

The multicentre cohort studies were built on standardized common protocols as well as a series of SOP to ensure systematic and homogenous execution of clinical and laboratory study procedures and sampling. Harmonisation of protocols for clinical cohorts as well as diagnostic algorithms has been ongoing between members of the ZIKAlliance consortium and other currently active research programs, funded for example by the EC or by the National Institutes of Health (NIH). The benefit of this is that patients can be enrolled and studied outside of ZIKAlliance with compatible protocols, which were made publicly available [83], and a pooled dataset may later be analysed for the maximum benefit.

Clinical monitoring is integrated into the programme of work from the outset, ensuring compliance with Good Clinical Practice (GCP) standards. The studies are conducted in accordance with the International Conference on Harmonisation - GCP guidelines. A reciprocal clinical monitoring network was set up between the partners of the Consortium as part of preparedness and capacity building.

The observational studies outlined here are conducted in accordance with the principles of the Declaration of Helsinki, the Nagoya protocol, GCP and relevant local regulations. All procedures employed in the clinical and related laboratory studies comply with local and international legislations regarding research involving human subjects, biobanking, and shipping of specimens. The protocols, amendments, consent and assent forms have been submitted to the relevant Research Ethics Committees at each study site, and to relevant regulatory authorities for written approval.

In the case of a clinical episode suggestive of ZIKV, the national standard of care and visitation schedule will be followed. The study team is in close contact with national authorities and will inform the surveillance teams about trends of confirmed infections per site. Unexpected incidental findings (not related to ZIKV or other arboviral infections) may occasionally be identified in individual patients in the course of the clinical study. The patient and/or parent/guardian will be informed, and with their consent, a referral will be made to an appropriate clinic or health facility for further investigation or longer-term follow-up.

Study results will be fed back to participants through periodic reports, and relevant results will also be communicated to healthcare professionals and health authorities. Scientific dissemination will not make use of professional writers and publications resulting from the study will use prevailing authorship eligibility guidelines [84]. It will be stated that the EC has no role on study design, data collection, analysis, interpretation of results, manuscript writing or decision to publish resulting reports.

## Discussion

After 2018, ZIKV transmission is projected to be low in the major urban areas of Latin America [85] until susceptibles have built up after approximately 10 years [5]. In the meantime, the results of these large cohort studies will provide better risk estimates for birth defects and other developmental abnormalities associated with ZIKV infections including possible co-factors that explain the variability of risk estimates currently seen between the Northeast of Brazil and other countries and regions. The ZIKAlliance Consortium covers a broad geographic region in Latin America and the Caribbean in order to represent the different environmental and demographic conditions that may influence the development and outcome of the ZIKV epidemic. These data will help decision-makers to allocate prevention and health care resources according to severity and impact of the epidemic. In addition, the results will provide a historical baseline for future intervention trials, should a vaccine or therapeutics become available and be tested without a control arm, as has recently been done in Ebola vaccine trials [86].

## Data Availability

The datasets used and/or analyzed during this study will be available from the corresponding author on reasonable request.

## Abbreviations

CH: Children
CHIKV: Chikungunya
CRFs: Case report forms
CZS: Congenital Zika syndrome
DENV: Dengue virus
ECLAMC: Latin American Collaborative Study on Congenital Malformations
EC: European Commission
EQA: External Quality Assurance
GCP: Good Clinical Practice
HH: Household
NH: Natural history
NIH: National Institutes of Health
PW: Pregnant women
RT-PCR: Reverse transcriptase PCR
TORCHS: Toxoplasmosis, Rubella, Cytomegalovirus, Herpes and Syphilis
VNT: Virus neutralisation
WHO: World Health Organization
WPs: Work packages
YF: Yellow fever
ZIKV: Zika virus

## References

[1] Dick GW, Kitchen SF, Haddow AJ (1952). Zika virus. I. Isolations and serological specificity. Trans R Soc Trop Med Hyg.

[2] Dick GW (1953). Epidemiological notes on some viruses isolated in Uganda; yellow fever, Rift Valley fever, Bwamba fever, West Nile, Mengo, Semliki forest, Bunyamwera, Ntaya, Uganda S and Zika viruses. Trans R Soc Trop Med Hyg.

[3] Dick GW (1952). Zika virus. II. Pathogenicity and physical properties. Trans R Soc Trop Med Hyg.

[4] Fauci AS, Morens DM (2016). Zika virus in the Americas--yet another Arbovirus threat. N Engl J Med.

[5] Ferguson N. M., Cucunuba Z. M., Dorigatti I., Nedjati-Gilani G. L., Donnelly C. A., Basanez M.-G., Nouvellet P., Lessler J. (2016). Countering the Zika epidemic in Latin America. Science.

[6] Parra B, Lizarazo J, Jimenez-Arango JA, Zea-Vera AF, Gonzalez-Manrique G, Vargas J, Angarita JA, Zuniga G, Lopez-Gonzalez R, Beltran CL (2016). Guillain-Barre syndrome associated with Zika virus infection in Colombia. N Engl J Med.

[7] Pacheco O, Beltran M, Nelson CA, Valencia D, Tolosa N, Farr SL, Padilla AV, Tong VT, Cuevas EL, Espinosa-Bode A, et al. Zika virus disease in Colombia - preliminary report. N Engl J Med. 2016. 10.1056/NEJMoa1604037. 10.1056/NEJMoa1604037 27305043

[8] Brasil P, Calvet GA, Siqueira AM, Wakimoto M, de Sequeira PC, Nobre A, Quintana Mde S, Mendonca MC, Lupi O, de Souza RV (2016). Zika virus outbreak in Rio de Janeiro, Brazil: clinical characterization, epidemiological and Virological aspects. PLoS Negl Trop Dis.

[9] Schuler-Faccini L, Ribeiro EM, Feitosa IM, Horovitz DD, Cavalcanti DP, Pessoa A, Doriqui MJ, Neri JI, Neto JM, Wanderley HY (2016). Possible association between Zika virus infection and microcephaly - Brazil, 2015. MMWR Morb Mortal Wkly Rep.

[10] Teixeira MG, Costa Mda C, de Oliveira WK, Nunes ML, Rodrigues LC (2016). The epidemic of Zika virus-related microcephaly in Brazil: detection, control, etiology, and future scenarios. Am J Public Health.

[11] Broutet N, Krauer F, Riesen M, Khalakdina A, Almiron M, Aldighieri S, Espinal M, Low N, Dye C (2016). Zika virus as a cause of neurologic disorders. N Engl J Med.

[12] Cao-Lormeau Van-Mai, Blake Alexandre, Mons Sandrine, Lastère Stéphane, Roche Claudine, Vanhomwegen Jessica, Dub Timothée, Baudouin Laure, Teissier Anita, Larre Philippe, Vial Anne-Laure, Decam Christophe, Choumet Valérie, Halstead Susan K, Willison Hugh J, Musset Lucile, Manuguerra Jean-Claude, Despres Philippe, Fournier Emmanuel, Mallet Henri-Pierre, Musso Didier, Fontanet Arnaud, Neil Jean, Ghawché Frédéric (2016). Guillain-Barré Syndrome outbreak associated with Zika virus infection in French Polynesia: a case-control study. The Lancet.

[13] World Health Organization, “WHO Director-General summarizes the outcome of the Emergency Committee regarding clusters of microcephaly and Guillain-Barré syndrome” (WHO, Geneva, 2016); http://bit.ly/HealthEmergency. In*.*

[14] Jaenisch T, Tam DT, Kieu NT, Van Ngoc T, Nam NT, Van Kinh N, Yacoub S, Chanpheaktra N, Kumar V, See LL (2016). Clinical evaluation of dengue and identification of risk factors for severe disease: protocol for a multicentre study in 8 countries. BMC Infect Dis.

[15] Messina JP, Kraemer MU, Brady OJ, Pigott DM, Shearer FM, Weiss DJ, Golding N, Ruktanonchai CW, Gething PW, Cohn E, et al. Mapping global environmental suitability for Zika virus. Elife. 2016;5:e15272. 10.7554/eLife.15272 PMC4889326 27090089

[16] Netto EM, Moreira-Soto A, Pedroso C, Hoser C, Funk S, Kucharski AJ, Rockstroh A, Kummerer BM, Sampaio GS, Luz E, et al. High Zika Virus Seroprevalence in Salvador, Northeastern Brazil Limits the Potential for Further Outbreaks. MBio. 2017;8(6):e01390-17. 10.1128/mBio.01390-17 PMC5686533 29138300

[17] Magalhaes T, Braga C, Cordeiro MT, Oliveira ALS, Castanha PMS, Maciel APR, Amancio NML, Gouveia PN, Peixoto-da-Silva VJ, Peixoto TFL (2017). Zika virus displacement by a chikungunya outbreak in Recife, Brazil. PLoS Negl Trop Dis.

[18] Saba Villarroel PM, Nurtop E, Pastorino B, Roca Y, Drexler JF, Gallian P, Jaenisch T, Leparc-Goffart I, Priet S, Ninove L (2018). Zika virus epidemiology in Bolivia: a seroprevalence study in volunteer blood donors. PLoS Negl Trop Dis.

[19] Brasil P, Pereira JP, Moreira ME, Ribeiro Nogueira RM, Damasceno L, Wakimoto M, Rabello RS, Valderramos SG, Halai UA, Salles TS (2016). Zika virus infection in pregnant women in Rio de Janeiro. N Engl J Med.

[20] Cauchemez S, Besnard M, Bompard P, Dub T, Guillemette-Artur P, Eyrolle-Guignot D, Salje H, Van Kerkhove MD, Abadie V, Garel C (2016). Association between Zika virus and microcephaly in French Polynesia, 2013-15: a retrospective study. Lancet.

[21] de Araujo TVB, Ximenes RAA, Miranda-Filho DB, Souza WV, Montarroyos UR, de Melo APL, Valongueiro S, de Albuquerque M, Braga C, Filho SPB (2018). Association between microcephaly, Zika virus infection, and other risk factors in Brazil: final report of a case-control study. Lancet Infect Dis.

[22] Mlakar J, Korva M, Tul N, Popovic M, Poljsak-Prijatelj M, Mraz J, Kolenc M, Resman Rus K, Vesnaver Vipotnik T, Fabjan Vodusek V (2016). Zika virus associated with microcephaly. N Engl J Med.

[23] Martines Roosecelis Brasil, Bhatnagar Julu, de Oliveira Ramos Ana Maria, Davi Helaine Pompeia Freire, Iglezias Silvia D'Andretta, Kanamura Cristina Takami, Keating M Kelly, Hale Gillian, Silva-Flannery Luciana, Muehlenbachs Atis, Ritter Jana, Gary Joy, Rollin Dominique, Goldsmith Cynthia S, Reagan-Steiner Sarah, Ermias Yokabed, Suzuki Tadaki, Luz Kleber G, de Oliveira Wanderson Kleber, Lanciotti Robert, Lambert Amy, Shieh Wun-Ju, Zaki Sherif R (2016). Pathology of congenital Zika syndrome in Brazil: a case series. The Lancet.

[24] Sheridan MA, Yunusov D, Balaraman V, Alexenko AP, Yabe S, Verjovski-Almeida S, Schust DJ, Franz AW, Sadovsky Y, Ezashi T (2017). Vulnerability of primitive human placental trophoblast to Zika virus. Proc Natl Acad Sci U S A.

[25] Cugola FR, Fernandes IR, Russo FB, Freitas BC, Dias JL, Guimaraes KP, Benazzato C, Almeida N, Pignatari GC, Romero S (2016). The Brazilian Zika virus strain causes birth defects in experimental models. Nature.

[26] Li C, Xu D, Ye Q, Hong S, Jiang Y, Liu X, Zhang N, Shi L, Qin CF, Xu Z (2016). Zika virus disrupts neural progenitor development and leads to microcephaly in mice. Cell Stem Cell.

[27] Dudley DM, Aliota MT, Mohr EL, Weiler AM, Lehrer-Brey G, Weisgrau KL, Mohns MS, Breitbach ME, Rasheed MN, Newman CM (2016). A rhesus macaque model of Asian-lineage Zika virus infection. Nat Commun.

[28] Olagnier D, Muscolini M, Coyne CB, Diamond MS, Hiscott J (2016). Mechanisms of Zika virus infection and Neuropathogenesis. DNA Cell Biol.

[29] Jaenisch T, Rosenberger KD, Brito C, Brady O, Brasil P, Marques ET (2017). Risk of microcephaly after Zika virus infection in Brazil, 2015 to 2016. Bull World Health Organ.

[30] Krauer F, Riesen M, Reveiz L, Oladapo OT, Martinez-Vega R, Porgo TV, Haefliger A, Broutet NJ, Low N, Group WHOZCW (2017). Zika virus infection as a cause of congenital brain abnormalities and Guillain-Barre syndrome: systematic review. PLoS Med.

[31] World Health Organization. Zika virus infection: update on the evidence for a causal link to congenital brain abnormalities and Guillain-Barré syndrome. Update of WHO Statement published on 31 March 2016. In*.*; 2016.

[32] Moore CA, Staples JE, Dobyns WB, Pessoa A, Ventura CV, Fonseca EB, Ribeiro EM, Ventura LO, Neto NN, Arena JF (2017). Characterizing the pattern of anomalies in congenital Zika syndrome for pediatric clinicians. JAMA Pediatr.

[33] Cleton NB, Godeke GJ, Reimerink J, Beersma MF, Doorn HR, Franco L, Goeijenbier M, Jimenez-Clavero MA, Johnson BW, Niedrig M (2015). Spot the difference-development of a syndrome based protein microarray for specific serological detection of multiple flavivirus infections in travelers. PLoS Negl Trop Dis.

[34] Wong SJ, Furuya A, Zou J, Xie X, Dupuis AP, Kramer LD, Shi PY (2017). A multiplex microsphere immunoassay for Zika virus diagnosis. EBioMedicine.

[35] Culjat M, Darling SE, Nerurkar VR, Ching N, Kumar M, Min SK, Wong R, Grant L, Melish ME (2016). Clinical and imaging findings in an infant with Zika Embryopathy. Clin Infect Dis.

[36] Cavalheiro S, Lopez A, Serra S, Da Cunha A, da Costa MD, Moron A, Lederman HM (2016). Microcephaly and Zika virus: neonatal neuroradiological aspects. Childs Nerv Syst.

[37] Cuevas EL, Tong VT, Rozo N, Valencia D, Pacheco O, Gilboa SM, Mercado M, Renquist CM, Gonzalez M, Ailes EC (2016). Preliminary report of microcephaly potentially associated with Zika virus infection during pregnancy - Colombia, January-November 2016. MMWR Morb Mortal Wkly Rep.

[38] Ventura CV, Maia M, Bravo-Filho V, Gois AL, Belfort R (2016). Zika virus in Brazil and macular atrophy in a child with microcephaly. Lancet.

[39] Ventura CV, Maia M, Dias N, Ventura LO, Belfort R (2016). Zika: neurological and ocular findings in infant without microcephaly. Lancet.

[40] Mehrjardi MZ. · Carteaux, G., · Poretti, a.,·, Taheri MS, · Bermudez, S., · Werner, H., · Hygino da Cruz Jr, L.C.: Neuroimaging findings of postnatally acquired Zika virus infection: a pictorial essay. Jpn J Radiol. 2017;35:341–9. 10.1007/s11604-017-0641-z 28447317

[41] van der Linden V, Pessoa A, Dobyns W, Barkovich AJ, Junior HV, Filho EL, Ribeiro EM, Leal MC, Coimbra PP, Aragao MF (2016). Description of 13 infants born during October 2015-January 2016 with congenital Zika virus infection without microcephaly at birth - Brazil. MMWR Morb Mortal Wkly Rep.

[42] Squires J, Bricker D (2009). Ages & stages questionnaires, third edition (ASQ-3): a parent completed child monitoring system.

[43] Bayley N (2006). Bayley scales of infant and toddler development 3rd edition (Bayley-III).

[44] de Oliveira WK, Carmo EH, Henriques CM, Coelho G, Vazquez E, Cortez-Escalante J, Molina J, Aldighieri S, Espinal MA, Dye C (2017). Zika virus infection and associated neurologic disorders in Brazil. N Engl J Med.

[45] Centers for Disease Control and Prevention Zika virus disease and Zika virus infection 2016 case definition. 2016. Available at: https://wwwn.cdc.gov/nndss/conditions/zika/case-definition/2016/06/.

[46] Duffy MR, Chen TH, Hancock WT, Powers AM, Kool JL, Lanciotti RS, Pretrick M, Marfel M, Holzbauer S, Dubray C (2009). Zika virus outbreak on Yap Island, Federated States of Micronesia. N Engl J Med.

[47] Lanciotti RS, Kosoy OL, Laven JJ, Velez JO, Lambert AJ, Johnson AJ, Stanfield SM, Duffy MR (2008). Genetic and serologic properties of Zika virus associated with an epidemic, yap state, Micronesia, 2007. Emerg Infect Dis.

[48] Cao-Lormeau VM, Roche C, Teissier A, Robin E, Berry AL, Mallet HP, Sall AA, Musso D (2014). Zika virus, French polynesia, south pacific, 2013. Emerg Infect Dis.

[49] Tappe Dennis, Pérez-Girón José Vicente, Zammarchi Lorenzo, Rissland Jürgen, Ferreira Davis F., Jaenisch Thomas, Gómez-Medina Sergio, Günther Stephan, Bartoloni Alessandro, Muñoz-Fontela César, Schmidt-Chanasit Jonas (2015). Cytokine kinetics of Zika virus-infected patients from acute to reconvalescent phase. Medical Microbiology and Immunology.

[50] Anaya J-M, Ramirez-Santana C, Salgado-Castaneda I, Chang C, Ansari A, Gershwin ME (2016). Zika virus and neurologic autoimmunity: the putative role of gangliosides. BMC Med.

[51] Oehler E, Watrin L, Larre P, Leparc-Goffart I, Lastere S, valour F, Baudouin L, Mallet H, Musso D, Ghawche F (2014). Zika virus infection complicated by Guillain-Barre syndrome--case report, French Polynesia**,** December 2013. Euro Surveill.

[52] Mecharles S, Herrmann C, Poullain P, Tran TH, Deschamps N, Mathon G, Landais A, Breurec S, Lannuzel A (2016). Acute myelitis due to Zika virus infection. Lancet.

[53] Carteaux G, Maquart M, Bedet A, Contou D, Brugieres P, Fourati S, Cleret de Langavant L, de Broucker T, Brun-Buisson C, Leparc-Goffart I (2016). Zika virus associated with Meningoencephalitis. N Engl J Med.

[54] World Health Organization. (2016). Zika virus disease: interim case definitions. World Health Organization. Available at: https://apps.who.int/iris/handle/10665/204381.

[55] Barjas-Castro ML, Angerami RN, Cunha MS, Suzuki A, Nogueira JS, Rocco IM, Maeda AY, Vasami FG, Katz G, Boin IF (2016). Probable transfusion-transmitted Zika virus in Brazil. Transfusion.

[56] Musso D, Nhan T, Robin E, Roche C, Bierlaire D, Zisou K, Shan Yan A, Cao-Lormeau V, Broult J (2014). Potential for Zika virus transmission through blood transfusion demonstrated during an outbreak in French Polynesia, November 2013 to February 2014. Eurosurveillance.

[57] Lanteri MC, Kleinman SH, Glynn SA, Musso D, Keith Hoots W, Custer BS, Sabino EC, Busch MP (2016). Zika virus: a new threat to the safety of the blood supply with worldwide impact and implications. Transfusion.

[58] Hills SL, Russell K, Hennessey M, Williams C, Oster AM, Fischer M, Mead P (2016). Transmission of Zika virus through sexual contact with travelers to areas of ongoing transmission - continental United States, 2016. MMWR Morb Mortal Wkly Rep.

[59] Foy BD, Kobylinski KC, Chilson Foy JL, Blitvich BJ, Travassos da Rosa A, Haddow AD, Lanciotti RS, Tesh RB (2011). Probable non-vector-borne transmission of Zika virus, Colorado, USA. Emerg Infect Dis.

[60] McCarthy M (2016). Zika virus was transmitted by sexual contact in Texas, health officials report. BMJ.

[61] Venturi G, Zammarchi L, Fortuna C, Remoli ME, Benedetti E, Fiorentini C, Trotta M, Rizzo C, Mantella A, Rezza G, et al. An autochthonous case of Zika due to possible sexual transmission, Florence, Italy, 2014. Euro Surveill. 2016;21(8):30148. 10.2807/1560-7917.ES.2016.21.8.30148 26939607

[62] Freour T, Mirallie S, Hubert B, Splingart C, Barriere P, Maquart M, Leparc-Goffart I. Sexual transmission of Zika virus in an entirely asymptomatic couple returning from a Zika epidemic area, France, April 2016. Euro Surveill. 2016;21(23):30254. 10.2807/1560-7917.ES.2016.21.23.30254 27311680

[63] Frank C, Cadar D, Schlaphof A, Neddersen N, Gunther S, Schmidt-Chanasit J, Tappe D. Sexual transmission of Zika virus in Germany, April 2016. Euro Surveill. 2016;21(23):30252. 10.2807/1560-7917.ES.2016.21.23.30252 27311329

[64] Turmel JM, Abgueguen P, Hubert B, Vandamme YM, Maquart M, Le Guillou-Guillemette H, Leparc-Goffart I (2016). Late sexual transmission of Zika virus related to persistence in the semen. Lancet.

[65] Davidson A, Slavinski S, Komoto K, Rakeman J, Weiss D (2016). Suspected female-to-male sexual transmission of Zika virus - New York City, 2016. MMWR Morb Mortal Wkly Rep.

[66] Swaminathan S, Schlaberg R, Lewis J, Hanson KE, Couturier MR (2016). Fatal Zika virus infection with secondary nonsexual transmission. N Engl J Med.

[67] Matheron S, d'Ortenzio E, Leparc-Goffart I, Hubert B, de Lamballerie X, Yazdanpanah Y (2016). Long-lasting persistence of Zika virus in semen. Clin Infect Dis.

[68] Meaney-Delman D, Oduyebo T, Polen KN, White JL, Bingham AM, Slavinski SA, Heberlein-Larson L, St George K, Rakeman JL, Hills S (2016). Prolonged detection of Zika virus RNA in pregnant women. Obstet Gynecol.

[69] Driggers RW, Ho CY, Korhonen EM, Kuivanen S, Jaaskelainen AJ, Smura T, Rosenberg A, Hill DA, DeBiasi RL, Vezina G (2016). Zika virus infection with prolonged maternal Viremia and fetal brain abnormalities. N Engl J Med.

[70] Charrel RN, Leparc-Goffart I, Pas S, de Lamballerie X, Koopmans M, Reusken C (2016). Background review for diagnostic test development for Zika virus infection. Bull World Health Organ.

[71] Mogling R, Zeller H, Revez J, Koopmans M, group Zrl, Reusken C: Status, quality and specific needs of Zika virus (ZIKV) diagnostic capacity and capability in National Reference Laboratories for arboviruses in 30 EU/EEA countries, May 2016. Euro Surveill. 2017;22(36):30609. 10.2807/1560-7917.ES.2017.22.36.30609 PMC5685210 28920574

[72] Corman VM, Rasche A, Baronti C, Aldabbagh S, Cadar D, Reusken CB, Pas SD, Goorhuis A, Schinkel J, Molenkamp R (2016). Assay optimization for molecular detection of Zika virus. Bull World Health Organ.

[73] Charrel R, Mogling R, Pas S, Papa A, Baronti C, Koopmans M, Zeller H, Leparc-Goffart I, Reusken CB (2017). Variable sensitivity in molecular detection of Zika virus in European expert laboratories: external quality assessment, November 2016. J Clin Microbiol.

[74] Fischer C, Pedroso C, Mendrone A, Bispo de Filipis AM, Vallinoto ACR, Ribeiro B, Durignon EL, Marques ETA, Campos GS, Viana IFT, Levi JE, Scarpelli LE, Nogueira M, de Souza Bastos M, Souza NCS, Khouri R, Lira SMC, Komninakis SV, Baronti C, Charrel RN, Kümmerer BM, Drosten C, Brites C, de Lamballerie X, Niedrig M, Netto EM, Drexler JF (2018). External Quality Assessment for Zika Virus Molecular Diagnostic Testing, Brazil. Emerg Infect Dis.

[75] L'Huillier AG, Hamid-Allie A, Kristjanson E, Papageorgiou L, Hung S, Wong CF, Stein DR, Olsha R, Goneau LW, Dimitrova K (2017). Evaluation of Euroimmun anti-Zika virus IgM and IgG enzyme-linked Immunosorbent assays for Zika virus serologic testing. J Clin Microbiol.

[76] Harris PA, Taylor R, Thielke R, Payne J, Gonzalez N, Conde JG (2009). Research electronic data capture (REDCap)--a metadata-driven methodology and workflow process for providing translational research informatics support. J Biomed Inform.

[77] ECLAMC (2015). Frequencia de microdefalia ao nascimento no Brasil. Periodo 1982–2013.

[78] Honein MA, Dawson AL, Petersen EE, Jones AM, Lee EH, Yazdy MM, Ahmad N, Macdonald J, Evert N, Bingham A (2017). Birth defects among fetuses and infants of US women with evidence of possible Zika virus infection during pregnancy. JAMA.

[79] Reynolds MR, Jones AM, Petersen EE, Lee EH, Rice ME, Bingham A, Ellington SR, Evert N, Reagan-Steiner S, Oduyebo T (2017). Vital signs: update on Zika virus-associated birth defects and evaluation of all U.S. infants with congenital Zika virus exposure - U.S. Zika pregnancy registry, 2016. MMWR Morb Mortal Wkly Rep.

[80] Shapiro-Mendoza CK, Rice ME, Galang RR, Fulton AC, VanMaldeghem K, Prado MV, Ellis E, Anesi MS, Simeone RM, Petersen EE (2017). Pregnancy outcomes after maternal Zika virus infection during pregnancy - U.S. territories, January 1, 2016-April 25, 2017. MMWR Morb Mortal Wkly Rep.

[81] Hoen B, Schaub B, Funk AL, Ardillon V, Boullard M, Cabie A, Callier C, Carles G, Cassadou S, Cesaire R (2018). Pregnancy outcomes after ZIKV infection in French territories in the Americas. N Engl J Med.

[82] Jaenisch Thomas, Sakuntabhai Anavaj, Wilder-Smith Annelies (2013). Dengue Research Funded by the European Commission-Scientific Strategies of Three European Dengue Research Consortia. PLoS Neglected Tropical Diseases.

[83] Van Kerkhove MD, Reveiz L, Souza JP, Jaenisch T, Carson G, Broutet N (2016). Working group on ZHR: harmonisation of Zika virus research protocols to address key public health concerns. Lancet Glob Health.

[84] International Committee of Medical Journal Editors- ICMJE. Defining the role of authors and contributors. Available at: http://www.icmje.org/recommendations/browse/roles-and-responsibilities/defining-the-role-of-authors-and-contributors.html.

[85] O'Reilly KM, Lowe R, Edmunds WJ, Mayaud P, Kucharski A, Eggo RM, Funk S, Bhatia D, Khan K, Kraemer MUG (2018). Projecting the end of the Zika virus epidemic in Latin America: a modelling analysis. BMC Med.

[86] Henao-Restrepo AM, Camacho A, Longini IM, Watson CH, Edmunds WJ, Egger M, Carroll MW, Dean NE, Diatta I, Doumbia M (2017). Efficacy and effectiveness of an rVSV-vectored vaccine in preventing Ebola virus disease: final results from the Guinea ring vaccination, open-label, cluster-randomised trial (Ebola Ca Suffit!). Lancet.

